# 

**Published:** 1859-09

**Authors:** 


### RECEIPTS FOR THE JOURNAL, UP TO SEPT. 8, 1859.

**ILLINOIS.**

| | | |
|---|---|---|
| Dr. T. B. Dever, | vol. 2, 1859, | $2 |
| " J. R. Conklin, | " " | 2 |
| " R. C Hamill, | " " | 2 |
| " A. P. Finch, | " " | 2 |
| " Abner Hard, | " " | 2 |
| " Kittoe, | " " | 2 |
| " J. P. Anthony, | vol. 1, 1858, | 2 |

**INDIANA.**

| | | |
|---|---|---|
| Dr. R. D. Manzy, | vols. 1 & 2, 1858-9, | $4 |
| " L. H. Kennedy. | vol. 1, 1858, | 2 |

**IOWA.**

| | | |
|---|---|---|
| Dr. H. B. Tuttle, | vol. 2, 1859, | $2 |

**MICHIGAN.**

| | | |
|---|---|---|
| Dr. Wm. Upjohn, | vols. 1 & 2, 1858-9, | $4 |

